# Development of Argon Isotope Reference Standards for the U.S. Geological Survey

**DOI:** 10.6028/jres.111.025

**Published:** 2006-10-01

**Authors:** Archie P. Miiller

**Affiliations:** National Institute of Standards and Technology, Gaithersburg, MD 20899-8364

**Keywords:** argon isotope standards, K-Ar dating or potassium-argon dating, transportable pipette systems

## Abstract

The comparison of physical ages of geological materials measured by laboratories engaged in geochronological studies has been limited by the accuracy of mineral standards or monitors for which reported ages have differed by as much as 2 %. In order to address this problem, the U.S. Geological Survey is planning to calibrate the conventional ^40^Ar/^40^K age of a new preparation of an international hornblende standard labeled MMhb-2. The ^40^K concentration in MMhb-2 has already been determined by the Analytical Chemistry Division at NIST with an uncertainty of 0.2 %. The ^40^Ar concentration will be measured by the USGS using the argon isotope reference standards that were recently developed by NIST and are described in this paper. The isotope standards were constructed in the form of pipette/reservoir systems and calibrated by gas expansion techniques to deliver small high-precision aliquots of high-purity argon. Two of the pipette systems will deliver aliquots of ^38^Ar having initial molar quantities of 1.567 × 10^−10^ moles and 2.313 × 10^−10^ moles with expanded (*k* = 2) uncertainties of 0.058 % and 0.054 %, respectively. Three other pipette systems will deliver aliquots (nominally 4 × 10^−10^ moles) of ^40^Ar:^36^Ar artificial mixtures with similar accuracy and with molar ratios of 0.9974 ± 0.06 %, 29.69 ± 0.06 %, and 285.7 ± 0.08 % (*k* = 2). These isotope reference standards will enable the USGS to measure the ^40^Ar concentration in MMhb-2 with an expanded uncertainty of ≈ 0.1 %. In the process of these measurements, the USGS will re-determine the isotopic composition of atmospheric Ar and calculate a new value for its atomic weight. Upon completion of the USGS calibrations, the MMhb-2 mineral standard will be certified by NIST for its K and Ar concentrations and distributed as a Standard Reference Material (SRM). The new SRM and the NIST-calibrated transportable pipette systems have the potential for dramatically improving the accuracy of interlaboratory calibrations and thereby the measured ages of geological materials, by as much as a factor of ten.

## 1. Introduction

The ^40^Ar/^39^Ar method [1], which is a variation of the conventional ^40^Ar/^40^K technique [2], can determine geochronological ages^1^ relative to a given primary mineral standard (or monitor) to within ≈ 0.1 to 0.25 %. However, the reported ages for the currently available interlaboratory mineral monitors differ by as much as 2 % and this has limited the comparison of ages determined by different laboratories [3]. In order to address this problem, the US Geological Survey is planning to calibrate the conventional ^40^Ar/^40^K age of MMhb-2, which is a new preparation of an international hornblende from the McClure Mountain Syenite that will serve as a new high-accuracy mineral monitor. The ^40^K concentration in the MMhb-2 has been determined by the Analytical Chemistry Division at NIST with an uncertainty^2^ of 0.2 % [4]. The ^40^Ar concentration in MMhb-2 will be measured by USGS using the argon isotope reference standards that were recently developed by NIST as described in this paper.

The specific objective of the present work is to develop isotope reference standards in the form of pipette systems capable of delivering small high-purity aliquots of ^38^Ar and artificial mixtures of ^40^Ar/^36^Ar. Two pipette systems are needed to deliver aliquots in the range of 2 × 10^−10^ moles to 4 × 10^−10^ moles of ^38^Ar with the molar quantity known to within an uncertainty of 0.25 % or better. In addition, three systems are needed to deliver similar quantities of ^40^Ar/^36^Ar mixtures with ratios (nominally 1:1, 30:1, 300:1) known to a similar uncertainty. An additional pipette system filled with a similar quantity of air is required to re-determine the isotopic composition of atmospheric argon. Each system consists of a reservoir containing the requisite isotopic quantities of argon and an attached pipette to deliver the desired aliquots.

The experimental design and choice of instrumentation are essentially dictated by the very small size of the aliquots. The delivery of these aliquots, using the smallest available pipette of 0.1 cm^3^, requires the reservoirs be filled to known pressures^3^ between 5 Pa and 10 Pa. The delivery of similar quantities of the artificial mixtures of ^40^Ar/^36^Ar require fill pressures for ^36^Ar as slow as 0.02 Pa. Measurements of such low pressures with requisite accuracy are at the state of the art and, therefore, they presented the major challenge in this work.

The description of the present work is divided into three major activities: the design and construction of the calibration system including the pipette systems; the calibration of the system volumes; and the filling of the reservoirs with requisite molar quantities of the argon isotopes.

## 2. Design and Construction of the Calibration System

### 2.1 Argon Isotope Source Gases

The ^38^Ar was supplied by the USGS in three glass vials with break-seals, which had been prepared several years earlier by E. Schumacher at the Institute for Organic Chemistry, University of Bern, Switzerland. The quantity of ^38^Ar in each vial was nominally 0.1 cm^3^ at STP (4.5 × 10^−6^ moles) with ^38^Ar/^36^Ar and ^38^Ar/^40^Ar ratios > 100,000 and > 10,000, respectively. The three vials were labeled Batch 2.8.3.21, Batch 2.8.3.24, and Batch 2.8.3.25.

The ^40^Ar and ^36^Ar were supplied with respective nominal purities of 99.99 % and 99.9 % in small metal bottles by Isotek, Inc.^4^ Gas chromatographic (GC) analysis by the supplier of the ^40^Ar indicated a chemical purity of 99.99 % with detectable impurities of N_2_, CO, CO_2_, and total hydrocarbons (THC), each less than 0.002 %. A mass spectrometric (MS) analysis by the supplier indicated the isotopic enrichment to be 99.964 % ^40^Ar. The GC analysis of the ^36^Ar indicated a chemical purity of 99.88 % with detectable impurities of H_2_ < 0.1 %, N_2_ 0.0065 %, and CO_2_ and THC, each less than 0.002 %. The MS analysis indicted the isotopic enrichment to be 99.7 atom % ^36^Ar and 0.3 atom % ^38^Ar.

### 2.2 The Pipette Systems

The first step in constructing the pipette systems was to fabricate cylindrical reservoirs from Type 304 stainless steel, four with a nominal volume of 1000 cm^3^ and three with a nominal volume of 3000 cm^3^. The reservoirs were fabricated from seamless tubing and all interior surfaces were electropolished prior to welding in order to reduce surface sites for gas adsorption. The component parts were welded together using full-penetration welds and helium as the shield gas rather than argon to minimize the possibility of trapping argon in the welds.

The reservoirs were then sent to Physikalisch-Technische Bundensanstalt, Berlin, Germany where they were vacuum fired at 950 °C for 2 h to reduce the amount of gas trapped in the bulk metal. This process significantly reduces subsequent outgassing, primarily due to hydrogen.

The Dörflinger pipettes^4^ [5] were also fabricated from stainless steel (except for copper valve seats). Each pipette was designed with two tubulated ports, one terminated in a mini-conflat and the other suitable for butt-welding. All stainless steel parts were vacuum fired to 950 °C prior to assembly. The volume of each pipette was defined by a small hole through a steel block that was closed by tightening copper valve seats on threaded drivers using a recommended torque of 14.2 N·m (126 lbf·in). The manufacturer determined each pipette volume (see Table A2 in the Appendix) by weighing the mercury required to fill the pipette. Four of the pipettes had a nominal volume of 0.1 cm^3^ and two pipettes had a nominal volume of 0.3 cm^3^.

**Fig. 1:**
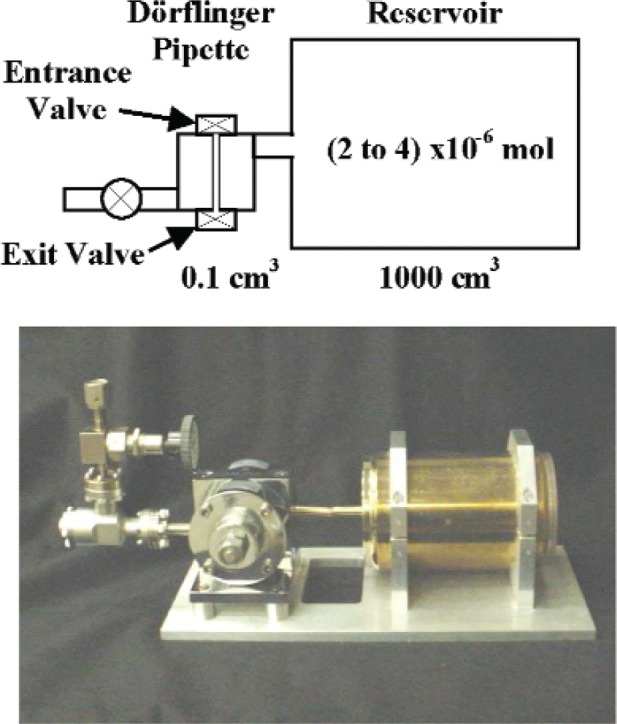
Schematic diagram/photograph of one of the pipette systems.

The final step in constructing the pipette systems involved butt-welding the pipettes to the reservoirs as shown in Fig. 1, using helium as the shield gas. All systems had pipette/reservoir volume ratios of nominally 1 × 10^−4^ except for one (a 0.1 cm^3^ pipette connected to a 3000 cm^3^ reservoir). The output of each pipette system was connected via the mini-conflat to another all-metal high vacuum valve. Besides being a safety feature, the additional valve serves as an auxiliary splitting system that can be used to size aliquots for the more sensitive mass spectrometers.

Prior to connecting the pipette systems into the calibration system, they were leak-tested by a “helium bombing” technique. The method utilized a helium leak detector to compare the 24 h accumulation of an evacuated pipette system placed in a helium atmosphere with the 24 h blanking accumulation of the pipette system in a vacuum environment. The (helium) leak rate was found to be < 10^−19^ mol/s for all pipette systems.

A large reference volume was constructed by butt-welding an all-metal bellows valve to the “extra” 1000-cm^3^ reservoir. This volume was calibrated gravimetrically with water (see Sec. 3.1) and then baked at high temperature to remove adsorbed water from the internal surfaces before it was connected into the calibration system.

### 2.3 Assembling and Baking the Calibration System

**Fig. 2:**
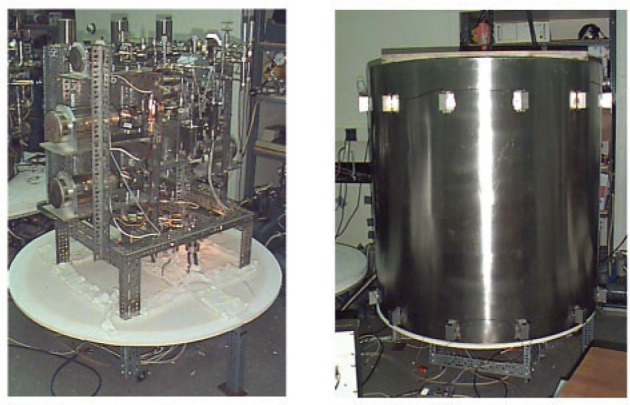
Photographs of the calibration system before baking (left) and enclosed in the high-temperature oven (right).

The calibration system was assembled by mounting the six pipette systems horizontally in two vertical groups of three and interconnecting them using all-metal high-vacuum plumbing as shown in Fig. 2 (a). Other components that were plumbed into the system included the large reference volume, a bakeable differential 10 Torr Capacitance Diaphragm Gauge (CDG), two metal seal-breaking devices containing ^38^Ar vials, two turbomolecular (TM) pumps for evacuating the system, and two Bayard-Alpert ionization gauges for monitoring the inlet pressures just above the TM pumps. A third Bayard-Alpert gauge was used to monitor the system base pressure near the top of the calibration system, furthest away from the TM pumps. This gauge was connected to the calibration system via an isolation valve so that it could be removed after baking.

Prior to baking, the all-metal valves (including the entrance and exit valves on the Dörflinger pipettes) were adjusted to their full open position to avoid possible damage to their valve seats during baking. Eight thermocouples were attached at distributed locations and a large cylindrical oven was lowered over the calibration system, as shown in Fig. 2 (b). A large disk fabricated from a low thermally conductive material (Marinite) was mounted on the lower part of the calibration system in order to enclose the heated volume of the oven.

The temperature inside the oven was slowly raised (0.2 °C/min) and maintained at 230 °C for a period of approximately 150 hours while pumping on the system. A system base pressure of ≈ 1 × 10^−6^ Pa (1 Pa = (1/133.3224) Torr) was achieved after cooling back to room temperature and the corresponding pressure above the inlets to the turbomolecular pumps was ≈ 4 × 10^−8^ Pa. The Bayard-Alpert gauge used to measure base pressure (at the top of the system) was then removed and all subsequent base pressures were estimated by multiplying the ionization gauge readings above the TM pumps by the above ratio, that is, 1 × 10^−6^/4 × 10^−8^ = 25.

**Fig. 3:**
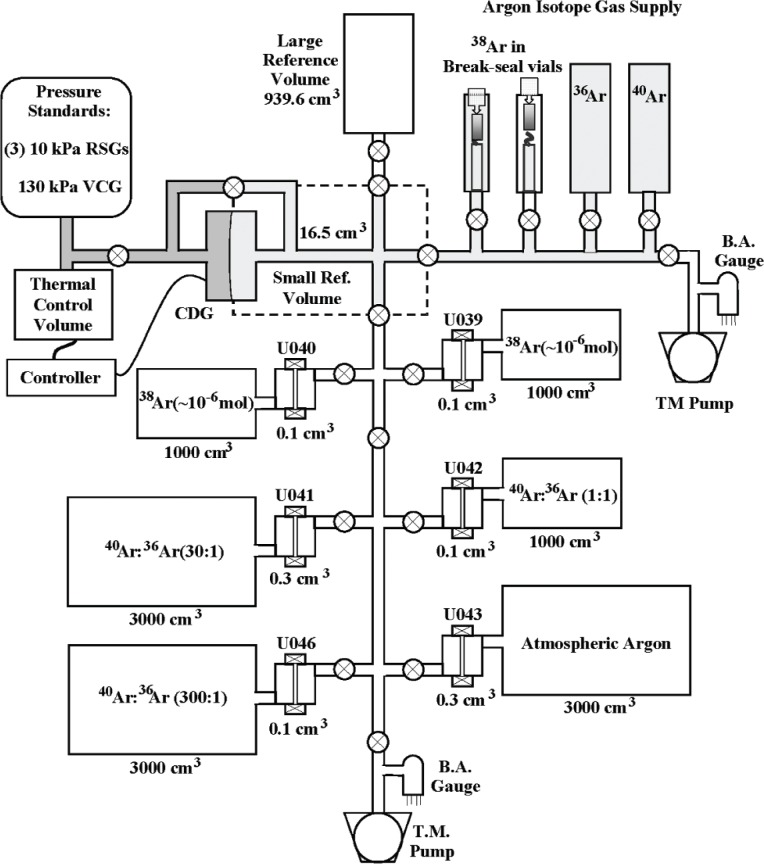
Schematic diagram of the calibration system used to measure volumes of the pipettes and reservoirs and to fill the reservoirs with requisite quantities of argon.

As the final step in assembly, two metal source bottles for ^40^Ar and ^36^Ar, a pressure controller and thermal control volume, and four high-accuracy pressure transducers were connected, in turn, to the calibration system. This required portions of the baked system to be reopened to the atmosphere. To minimize possible recontamination with atmospheric moisture the affected portion of the system was first isolated and back-filled with dry nitrogen, and then an outflow of nitrogen was maintained while the devices were connected into the system. The final configuration of the calibration system is shown schematically in Fig. 3, where the pressure standards consist of 3 RSGs (Resonant Silicon Gauges) and a VCG (Vibrating Cylinder Gauge). These gauges and the Thermal Control Volume are described in Sec. 2.4.3.

### 2.4 Measurement Techniques and Issues

Although NIST has exceptional pressure measurement capabilities, the effective use of these capabilities required specialized techniques and attention to detail in the measurements.

#### 2.4.1 General Approach

The desired small molar quantities can be achieved only with small volumes and low pressures. The values of all system volumes, large and small, were derived from a calibrated small reference volume (see Fig. 3) using gas expansion and the equation of state. This required the measurement of large pressure ratios and the accurate measurement of low pressures. The value of the small reference volume was calibrated by gas expansion into the large reference volume previously calibrated by a gravimetric method (Sec. 3.2).

Both volume determinations and reservoir fills utilized successive gas expansions from a small volume (*S*) into a “dead” or intermediate volume (*I*) and then into a large volume (*L*). Conservation of mass requires that the number of moles of gas in the system be conserved, i.e.,

$$n_{s0}=n_{s1}+n_{I1}=n_{sf}+n_{If}+n_{Lf}$$ (1)

where the subscripts 0, 1, and *f* refer to equilibrium conditions after the initial fill, the first expansion, and final expansion. The molar quantities in the volumes are related to pressure and temperature of the gas by the equation of state, *n* (*p*, *V*, *T*). For example, the initial number of moles in the small volume may be expressed as

$$n_{S0}=\frac{p_{0}V_{S}e_{S0}h_{S0}}{RT_{S0}z_{S0}}=\frac{p_{0}V_{S}C_{S0}}{RT_{S0}}$$ (2)

where

*V_S_* is the volume of gas in the small volume at 23 °C,
*t_S_*_0_ and *T_S_*_0_ are the Celsius and Kelvin temperatures of the gas,
*R* = 8.314472 J mol^−1^ K^−1^ is the molar gas constant [6],
*e_S_*_0_ = [1 + 3α (*t_S_*_0_ − 23)] is the volume expansion factor, α = 1.47 × 10^−5^ K^−1^ is the linear expansion coefficient for stainless steel [7],
*z_S_*_0_ = [1 + *B_V_ p*_0_/*R T_S_*_0_] is the compressibility factor for the equation of state [8] with the second virial coefficient^5^ *B_V_* = 5.49 cm^3^ mol^−1^ for nitrogen or 16.8 cm^3^ mol^−1^ for argon [9],
*h_S_*_0_ = [1 − *MgΔh_S_*/*RT_S_*_0_] is the gas head correction factor for pressure due to the difference in elevation (*Δh_S_*) of volume S relative to the CDG, which also served as the reference height for the pressure transducers (VCG and RSGs). The molar mass *M* = 28.014 g mol^−1^ for nitrogen and 39.948 g mol^−1^ for argon [10], and the local gravitational acceleration *g* = 9.80101 m s^−2^.

Similar equations describe the number of moles in the system volumes after the first and final expansions. The combined correction factors, such as, *C_S_*_0_ = *e_S_*_0_ *h_S_*_0_/*z_S_*_0_, were close to unity for most of the data taken during the calibrations.

These equations form the basis for calibrating the pipette and reservoir volumes as well as filling the reservoirs with the requisite molar quantities. If delivery of aliquots with the desired accuracy is to be achieved, several issues had to be considered to ensure reliable measurements of the equation-of-state parameters during the course of the calibrations.

#### 2.4.2 Molar Quantity

It is important that leaks, either real or virtual, especially of argon, are minimized and that outgassing from the inner surfaces of the reservoirs be minimal. Otherwise the total quantity of gas in the system volumes will not remain constant as assumed by Eq. (1), during the long time intervals required for thermal equilibrium. This can be accomplished by using Ultra High Vacuum techniques during the construction of the calibration system such as, full penetration welds, all metal seals, bellows or diaphragm all-metal valves, cleaning of system components, and bakeout to remove water from internal surfaces.

There are several issues that relate to the molar quantity of ^38^Ar in the pipette/reservoir systems. The first is physical adsorption of ^38^Ar by interior surfaces of the reservoirs and its possible effect on calibrations of molar quantities, which is discussed in Sec. 7. Other issues are related to experimental conditions that can affect accuracy of the aliquots of ^38^Ar at the time of use by the USGS. These issues are addressed in Sec. 5.

The possibility of an atmospheric leak into the reservoirs containing artificial mixtures after their calibration is another potential issue. Although atmospheric argon constitutes only 0.93 % of dry air by volume, it is primarily ^40^Ar (99.6 %) and a sizeable leak will affect the molar quantity of ^40^Ar and hence the precisely determined molar ratios. The upper limit to the leak rate for the pipette/reservoir systems was determined (see Sec. 2.2) as approximately 10^−19^ mol/s, which corresponds to an accumulation rate for atmospheric ^40^Ar of 3 × 10^−14^ moles per year. This is a negligible fraction of the calibrated molar quantities of ^40^Ar in the artificial mixtures as given in Table 1 of Sect. 5.

#### 2.4.3 Pressure

NIST has developed a group of Ultrasonic Interferometer Manometers (UIMs) as primary standards for the measurement of absolute pressure [11–13]. The mercury UIMs have full-scale ranges up to 360 kPa and a stated uncertainty of [(6 mPa)^2^ + (5.2 × 10^−6^ *p*)^2^]^1/2^ due to systematic effects, where *p* is the pressure measured in Pa. An oil UIM [14] with a full-scale range of 140 Pa was developed for low absolute pressures and has an uncertainty of [(3 mPa)^2^ + (36 × 10^−6^ *p*)^2^]^1/2^.

The mercury UIMs were not used to measure pressure directly in the present application largely because of the risk of contaminating the pipette systems with mercury. Instead, direct pressure measurements were made using high-accuracy pressure transducers as working standards, namely, three 10 kPa RSGs (resonant silicon gauges) for pressures between 100 Pa to 10 kPa and a 130 kPa VCG (vibrating cylinder gauge) for pressures above 10 kPa. The RSGs [15] are MEMS (Micro Electro Mechanical Systems) sensors that are manufactured by silicon micromachining techniques to produce silicon diaphragms nominally a few millimeters square by a fraction of a millimeter thick. Two single-crystal silicon resonators are encapsulated in vacuum microcavities that are micromachined onto the surface of the silicon diaphragm. Changes in pressure on the diaphragm are determined by measuring strain-induced changes in the two resonant frequencies. The two frequencies are used for temperature compensation to minimize thermal effects. The VCG utilizes a high-accuracy temperature-compensated 20 MHz crystal oscillator as a reference to measure changes in vibrational frequency of a cylindrical sensor in response to changes in density (pressure) of the gas inside the sensor.

The transducers were calibrated three times during the course of the present project by comparison with a mercury UIM. The uncertainties due to calibration instabilities were found to be about 0.01 % of the pressure reading for the RSGs and 0.001 % for the VCG.

The oil UIM was used to directly measure final fill pressures (3 Pa to 10 Pa) associated with molar quantity calibrations but these data were only useful for diagnostic purposes because the uncertainty at these low pressures was dominated by zero instabilities of the CDG (see below). Instead, the final fill pressures were calculated (with significantly greater accuracy) based on calibrated values for the system volumes and much higher initial pressures (≥ 235 Pa) in the small reference volume, which were measured by the RSGs.

The differential CDG (see Fig. 3) served two purposes. First, it was used to isolate the argon (or nitrogen) gas in the small reference volume from the nitrogen gas being used to transmit pressure to the VCG and RSGs. Second, it was used to measure the difference in pressure between the small reference volume and the pressure transducers, and to correct their readings. The corrections were minimized by maintaining the CDG readings near zero using active pressure control. The active control was based on a temperature-controlled volume of ≈ 50 cm^3^ (the Thermal Control Volume of Fig. 3), consisting of an array of 1-cm diameter holes drilled into a copper block. The temperature of the volume was controlled using a Peltier Heater/Cooler Module in thermal contact with the copper block, a bi-directional power supply, and an electronic controller employing both proportional and integral control. The controlling signal was generated by the difference between a stable adjustable voltage source and the output of the differential CDG. With active control, it was possible to achieve stable CDG readings of ± 10 mPa or less within minutes after gas additions to or gas expansions from the small reference volume.

The zero differential-pressure reading of the CDG will depend on line pressure, which in the present measurements ranged from a few Pascal to above an atmosphere. Auxiliary experiments were performed to measure the functional dependence of the CDG zero on line pressure yielding corrections to the CDG readings that were as large as 0.5 Pa at the highest pressures (≈ 130 kPa).

The performance of CDGs is limited at low pressures by two types of instabilities in their zero-pressure readings, those that correlate with changes in ambient temperature and those that vary randomly and are probably due to shifts in electronics and/or mechanical structure of the gauge [16]. To minimize temperature-induced shifts, the ambient temperature of the CDG was actively controlled by mounting the CDG inside a thick-walled (≈ 2 cm) aluminum housing in thermal contact with a Peltier heating/cooling module. An external power supply and bridge circuitry were used to control the current to the module in response to an auxiliary Platinum Resistance Thermometer (PRT) embedded in the housing. This arrangement controlled the ambient temperature of the CDG to within ± 3 mK, which corresponded to a temperature-induced zero-pressure instability that is about two orders of magnitude smaller than the 1 mPa resolution of the gauge.

The second type of zero instability is the likely source of CDG zero shifts observed in the present measurements. Gas additions to or gas expansions from the small reference volume were very delicate operations since compensating amounts of gas had to be simultaneously added to or removed from the backside of the differential CDG to avoid over-ranging, which could cause significant shifts in the CDG zero. The final expansions into the reservoirs involved large pressure excursions and were particularly susceptible to generating large zero shifts. Zero shifts were measured after evacuating the system at the conclusion of each set of volume or molar quantity calibrations and found to be in the range of −85 mPa to 70 mPa. These were used to correct pressures measured after the final expansions. The estimated uncertainty in making these corrections (± 15 mPa based on observed drifts in the CDG zero with its bypass open) is a very small fraction of the final pressures (≥ 700 Pa) associated with volume calibrations. However it is a significant fraction of the final pressures (3 Pa to10 Pa) associated with calibrations of molar quantities and so it limited the usefulness of direct measurements of pressure with the oil UIM.

#### 2.4.4 Volume

The small reference volume consisted of the volume associated with the interconnecting plumbing between four all-metal bellows valves when closed (valve seats facing inward) and the small volume in the front side of the CDG (≈ 2 cm^3^), as illustrated by the dashed lines in Fig. 3. The stability of its value (nominally 16.5 cm^3^) was influenced by three factors: deflection of the CDG diaphragm, deformation of the valve stem/seat during valve closure, and possible wear of the valve seat due to repeated closures. The first factor was minimized by the active pressure control used to keep the CDG reading near zero, which also maintained the CDG diaphragm in a near-zero deflection state. Repeatable deformations of the valve stems/seats were maintained by always closing, the four valves with the same small torque of 0.9 N·m (8 lbf·in). In order to check for evidence of wear in the valve seats, repeat calibrations of the small reference volume were performed before and after calibrating the volumes of the six pipette-reservoir systems.

The intermediate or “dead” volume (*V_I_*) was defined as the internal volume of the plumbing connecting the pipettes to the small reference volume (*V_S_*) with all interconnecting isolation bellows valves fully opened. In addition, *V_I_* included small volumes of the pipettes with their entrance valves (see Fig. 1) closed using a fixed torque of 14.2 N·m (126 lbf·in) and their exit valves turned to a repeatable open position using a torque of 3.4 N·m (30 lbf·in). Unlike the bellows-type valves, there is no positive stop when opening the exit/entrance valves of the pipette. Rather a rapid increase in torque is required as the “full” open position is approached.

For the ^38^Ar pipette-reservoir systems (see Fig. 3) *V_I_* was nominally 42 cm^3^, which included only the volumes of the pipette pair U039 and U040 by closing the isolation valve immediately below this system. This valve was fully opened for measurements on the other pipette systems and the intermediate volume, which included the volumes of all pipettes, was nominally 125 cm^3^.

The deformation of the pipette valve seats under applied torque deserves careful consideration. When calibrating the volume or the molar quantity of gas in a given reservoir, the pipette entrance valve is closed under the specified torque after gas expansion into the reservoir. However, the division of molar quantity of gas occurs at initial contact between the copper valve seat and the stainless steel valve body of the pipette. Further closure of the pipette entrance valve with the required torque will be accompanied by a small pressure rise in the small reference volume and intermediate volume due to penetration of the copper valve seat into the pipette thereby decreasing its volume (and hence that of the intermediate volume) by a small amount, δν. Since the number of moles of gas in the reservoir remains constant during the application of torque, the fill pressure that will be measured after closure may be expressed as

$$p_{f}=p_{f}{}^{\prime }[1+\delta \nu /(V_{S}+V_{I})]=p_{f}{}^{\prime }f$$ (3)

and the “effective” volume of the reservoir associated with *p_f_* is given by

$$V_{L}=V_{L}{}^{\prime }[1+\partial \nu /{\left(V_{S}+V_{I}\right)}^{-1}=V_{L}{}^{\prime }f^{-1}$$ (4)

where the primed quantities refer to values of parameters at initial valve seat contact. By using Eqs. (1), (2), and (4), the definition $V_{I}=V_{I}{}^{\prime }-\partial \nu$, and the simplifying assumption of uniform gas temperature, the molar division of gas in the system may be expressed to a good approximation in terms of the following volume ratios

$$\frac{n_{Lf}}{\left(n_{Sf}+n_{If}\right)}≅\frac{V_{L}^{\prime }}{\left(V_{S}+V_{I}^{\prime }\right)}=\frac{V_{L}}{\left(V_{S}+V_{I}\right)}$$ (5)

This illustrates that either set of parameters, (*p_f_*, *V_L_*, *V_I_*) or ( $p_{f}^{\prime }$, $V_{L}^{\prime }$, $V_{I}^{\prime }$), will give the correct molar division of gas. However, since $V_{I}^{\prime }$ cannot be measured directly and for other practical reasons, the volume and molar quantity calibrations of the reservoirs were based on parameters obtained with the pipette entrance valves closed under torque.

The volume of each pipette is defined by the method used to draw an aliquot of gas from the reservoir. First the exit valve of the evacuated pipette is closed using a torque of 14.2 N·m. The entrance valve is opened to fill the pipette and then closed slowly until initial valve seat contact is made. The volume (ν*_D_*′) of the Dörflinger pipette and its molar content is established at this point. If the gas in the pipette and the reservoir are at the same temperature and pressure, then the pipette to reservoir volume ratio at initial valve seat contact determines the ratio of molar quantity of gas in the pipette (*n_D_*) to that in the reservoir (*n_Lf_*), i.e.,

$$r_{V}=\frac{\nu_{D}{}^{\prime }}{V_{L}^{\prime }}=\frac{\nu_{D}{}^{\prime }}{V_{L}f}=\frac{n_{D}}{n_{Lf}}$$ (6)

Since it is *V_L_* and not $V_{L}^{\prime }$ that was determined in calibrating a reservoir volume, the valve seat deformation *δν* is needed to determine the factor *f* as defined by Eq. (4) and hence *r_V_*.

In order to study valve seat deformation, an auxiliary experiment was performed on an available 0.1-cm^3^ pipette using an experimental setup similar to the calibration system shown in Fig. 3 but without the burden of gas supply bottles/vials and pipette/reservoir systems. One side of the pipette (with its valve open) was connected directly to the small “reference” volume while the other side of the pipette was capped. The small volume, *V_S_*, and intermediate volume, *V_I_*, were calibrated using techniques described in Secs. 3.2 and 3.3. The deformation was determined by solving Eq. (4) for δν and measuring the pressure rise, $p_{f}-p_{f}^{\prime }$, in the small and intermediate volumes as a function of closing torque applied to the capped-side valve. The measurements were performed at a nominal pressure of 130 kPa using the VCG and active pressure control to maintain the CDG near zero deflection. The connections of the pipette valves were reversed and the measurements were repeated on the second valve.

The average pipette volume decrease was determined as δν = (0.0052 ± 0.0003) cm^3^ when closing a single valve with an applied torque of 14.2 N·m. The corresponding values for *f* were then calculated as 1.000089 for calibration of the ^38^Ar systems (V_I_ = 42 cm^3^) and 1.000037 for the artificial mixture systems (V_I_ = 125 cm^3^)

The functional dependence on closing torque τ was also determined by least-squares fitting the following functions to data on distortion versus applied torque:

$$\begin{array}{l} \delta \nu =7.99\times 10^{-5}\tau +3.65\times 10^{-5}\tau^{2}-1.16\times 10^{-6}\tau^{3}\;\tau \;\text{in}\;N\cdot m\\ \text{or}\\ \delta \nu =9.03\times 10^{-6}\tau +4.66\times 10^{-7}\tau^{2}-1.67\times 10^{-9}\tau^{3}\;\tau \;\text{in}\;\text{lbf}\cdot \text{in} \end{array}$$ (7)

The pipette volume will be very sensitive to any deviation from the prescribed applied torque when closing the exit valve. The torque wrench used in the present work has a resolution of about 0.05 N·m (0.5 lbf·in), which corresponds to a resolution in determining the pipette volume and aliquot size of 0.025 %.

During calibrations, the large reference and reservoir volumes were subjected to a range of pressure gradients between their exterior and interior surfaces, from a few Pa to an atmosphere. In order to estimate the volume compressibility, the large reference volume was mounted inside a slightly larger steel container, which was then filled with water and closed to the atmosphere except for a small graduated capillary tube. By pressurizing or evacuating the reference volume it was possible to measure the changes in its volume from the displacement of water in the capillary. The measurements indicated a volume change of 0.0017 % for a pressure difference of 100 kPa, or a volume compressibility of (1/*V*)(Δ*V*/Δ*p*) = 1.7 × 10^−10^ Pa^−1^. This was used to correct the values of the large reference and reservoir volumes for different interior pressures used during calibrations.

#### 2.4.5 Temperature

There were significant temperature non-uniformities within the calibration system, largely because of its large size. The temperatures were measured using 100-ohm Platinum Resistance Thermometers (PRTs) mounted in small aluminum blocks, which were clamped to the calibration system at strategic locations. A total of nineteen PRTs were used and were mounted as follows: two PRTs on the large reference volume, one PRT on the intermediate volume between the large and small reference volumes, four PRTs on the small reference volume (including one in the CDG housing), and for each of the six pipette systems, one PRT on the Dörflinger pipette and another on the associated reservoir. The PRTs were calibrated by comparison to four standard PRTs that had been calibrated on ITS-90 [17] by the Temperature Group in the Process Measurements Division at NIST.

The PRTs only provide an indirect measure of the actual temperature of the gas at various points in the system. The transfer of gas from a volume at high pressure to a volume with much lower pressure or a vacuum is approximately adiabatic. The gas that is injected into the vessel becomes much warmer than the surrounding walls while the gas remaining on the high-pressure side, which is performing the work, cools down considerably below ambient temperature. The magnitude of the temperature changes and the time constants associated with relaxation of the gas temperature to that of the surrounding walls depends on details of the particular system [18]. In the present calibrations, the method for detecting thermal equilibrium was based on calculating the total number of moles of gas in the volumes as a function of time using data on the pressure of the gas and the mean temperature of the volume walls as measured by the PRTs. It was assumed that thermal equilibrium is achieved when the calculated number of moles no longer decreased (or increased) with time, indicating that the gas and volume walls had reached essentially the same temperature.

**Fig. 4:**
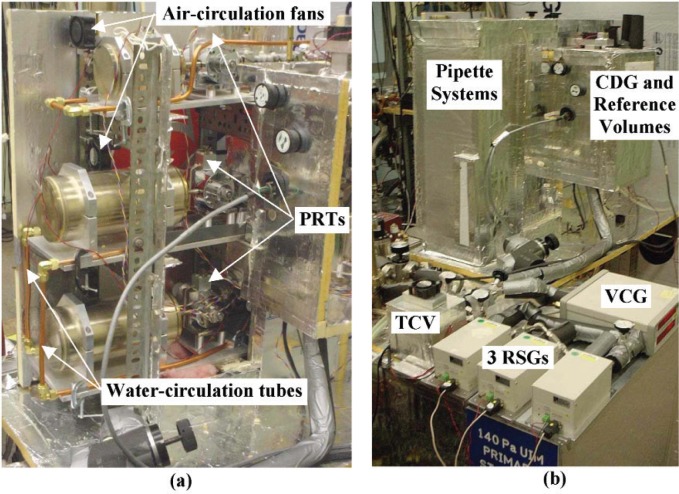
Photographs illustrating (a) methods used to measure and passively control temperature in the calibration system and (b) the calibration system with the RSGs, the VCG and the TCV.

Considerable time and effort were devoted to studying and reducing the influence of fluctuations in laboratory temperature on temperatures being measured in the calibration system. It was important that any lag between the temperature measured by the PRTs and the actual gas temperature was minimized as much as possible. Ultimately four different but complimentary measures were implemented to passively control temperatures in the system. Water from a temperature bath operating at 23.0 °C was circulated via copper tubing clamped to the base of each pipette system and to the large and small reference volumes [see Fig. 4(a)]. “Muffin” fans, which were operated at the lowest possible speed to minimize heat generated by their motors, were mounted throughout the system to circulate air in order to reduce temperature gradients. Sealed plastic bags filled with water were placed throughout the system in order to provide additional thermal inertia and thereby reduce the influence of rapid fluctuations in laboratory temperature. Finally, the pipette systems, the CDG, and the large and small reference volumes were entirely enclosed using 25 mm thick extruded-foam shielding with aluminum foil backing [see Fig. 4(b)].

## 3. Calibration of System Volumes

### 3.1 The Large Reference Volume

As mentioned earlier, the large reference volume was calibrated by a gravimetric method. The first step involved filling a 2000 cm^3^ stainless steel source reservoir with distilled water and pumping on it to remove any dissolved gases. The degassed water was then transferred under vacuum through a sighting tube attached to the open entrance valve of the reference volume. The reference volume was filled under vacuum to avoid air pockets, which would result in underfilling. After closing an entrance valve to the sighting tube, the filled reference volume plus sighting tube were placed in an air-bath operating at 23 °C for equilibration over an extended period, usually overnight. After measuring the temperature of the reference volume by means of three PRTs, the entrance valve to the sighting tube was opened momentarily to pressurize the water to one atmosphere. This process was usually accompanied by some reduction in height of the water in the sighting tube, possibly due to the filling of voids in the reference volume and/or collapsing of residual bubbles that had not been removed earlier. The valve to the reference volume was then closed and the sighting tube removed followed by pumping on the valve to remove any traces of water.

The weighing of the reference volume, both when empty (evacuated) and when filled, was carried out using the measurement protocol and the 5 kg Mass Comparator of the Mass Group in the Manufacturing Metrology Division of NIST. The correction for buoyancy of the air displaced by the reference volume was based on measurements of its air displacement volume, barometric pressure, relative humidity, air temperature, and data on density of moist air given in the literature [19,20]. The source of largest uncertainty in the buoyancy correction was the air displacement volume, which was known only to within 4.5 %. However since essentially the same correction is applied when weighing the volume empty or filled, the effect essentially cancels and the net uncertainty propagated [21] to the measured value of the large reference volume was less than 0.0002 %.

In each calibration, the volume was calculated by subtracting the mean of four determinations of the empty volume mass from the mass of the filled volume and dividing the result by the density of water [22] at the mean temperature reading of three PRTs attached to the volume. In each case the value of the reference volume was corrected to 23.0 °C using the linear thermal expansion coefficient for stainless steel (1.47 × 10^−5^ K^−1^). The mean value of the large reference volume at 23.0 °C for eight calibrations was 939.642 cm^3^ with an estimated uncertainty of 0.022 cm^3^ or 0.0023 %. The largest contributions to uncertainty were due to random scatter in four repeat calibrations of the empty reference volume (0.0015 %) and the scatter in eight repeat calibrations of the filled reference volume (0.0014 %). The source of this scatter is believed to be variable absorption of atmospheric moisture by the Phenolic handle on the reference volume entrance valve. Other estimated contributions to uncertainty were from dissolved air in water (0.0008 % [23]), variation in isotopic composition of water (0.0004 % [24]), PRT calibration (0.0004 %), and literature values for density of water (0.0002 % [22]).

### 3.2 The Small Reference Volume

#### 3.2.1 Basic Method

The calibration of the small reference volume followed essentially four steps. (1) The small volume was filled with nitrogen and, after sufficient time for equilibration, the initial gas pressure (*p*_0_) and the volume temperature were measured. (2) The gas was then expanded into the intermediate volume and, after equilibration, the gas pressure (*p*_1_) and the temperatures of both small and intermediate volumes were measured. (3) Finally the gas was expanded into the calibrated large reference volume and, after equilibration, the gas pressure (*p_f_*) and the temperatures of the small, intermediate, and large volumes were measured. The underlying assumption was that, after a sufficiently long equilibration time, the temperature of the volume walls would approximate the actual temperature of the gas contained therein. (4) The unknown volume was then calculated from the pressure and temperature data using Equation (1), which is actually two equations that can be solved to express the unknown small reference volume *V_S_* in terms of the known large reference volume *V_L_* following the nomenclature established in Equation (2):

$$V_{S}=\frac{V_{L}(p_{f}C_{Lf}/T_{Lf})}{\left[p_{0}C_{S0}/T_{S0}-p_{f}C_{Sf}/T_{Sf}-(p_{0}C_{S0}/T_{S0}-p_{1}C_{S1}/T_{S1})p_{f}C_{If}T_{I}{}_{1}/p_{1}C_{I}{}_{1}T_{If}\right]}$$ (8)

#### 3.2.2 Measurements

Before any meaningful measurements were made, the calibration system was evacuated for a sufficient time, sometimes several days depending on its previous history, to achieve a base pressure in the range of 3 × 10^−5^ Pa to 8 × 10^−5^ Pa (2 × 10^−7^ Torr to 6 × 10^−7^ Torr). Also, to ensure a stable small reference volume, the four valves that define it (see Fig. 3) were closed always with the same small torque of 0.9 N·m (8 lbf·in).

**Fig. 5:**
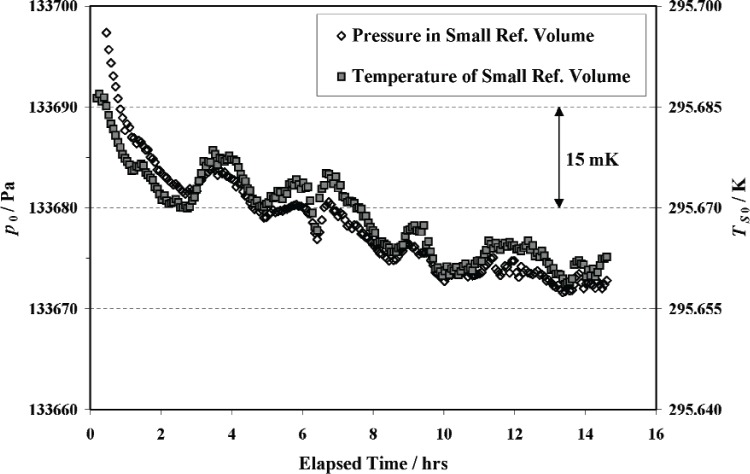
Variation of the pressure of nitrogen gas in the small reference volume and the volume temperature with time after an initial fill.

With the system evacuated, the CDG bypass was opened and the CDG was adjusted to read zero. Nitrogen gas was added to the small reference volume through the CDG bypass valve, which was then closed after achieving the desired fill pressure (≈ 130 kPa). The system was allowed to thermally equilibrate for about 15 h while readings of gas pressure and volume temperature were taken at 5-minute intervals. Figure 5 shows the pressure and temperature as a function of elapsed time. The variation of these quantities was in response to fluctuations in laboratory temperature, although after approximately 10 h the variation in volume temperature was only ± 2 mK and the variation in pressure, which directly follows the gas temperature, was only ± 2 Pa. For comparison, the corresponding variation in laboratory temperature was approximately ± 50 mK. It is not clear from this plot as to when or if thermal equilibrium was achieved, however, a more definitive method was used as described below.

The nitrogen gas was then expanded into the intermediate volume between the small and large volumes and allowed to equilibrate for approximately 6 h before final expansion into the large volume and equilibrating for another 12 h. Again data on gas pressure and temperatures of the volumes were taken at 5 min intervals. The final system pressure was nominally 2300 Pa.

**Fig. 6:**
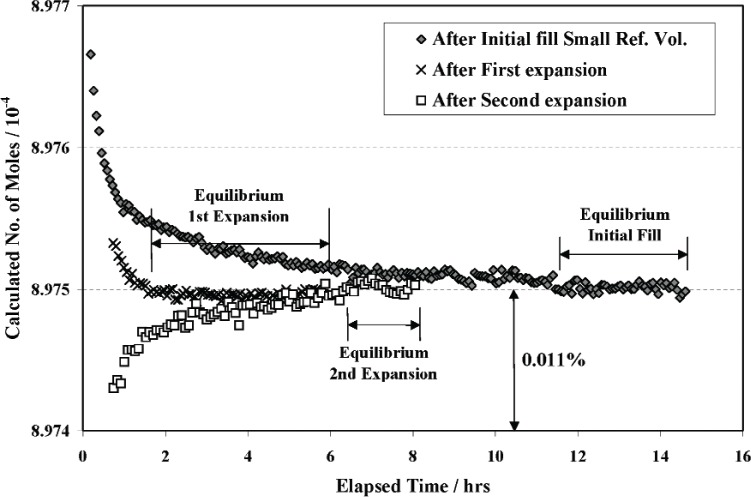
Calculated number of moles of nitrogen in the calibration system after the initial fill, after the first expansion, and after the second or final expansion. The time intervals over which equilibrium data were averaged are also shown.

Figure 6 shows the variation of the calculated number of moles with time after the initial fill, after the first expansion, and after the second expansion, where the number of moles after the initial fill corresponds to the pressure and temperature data shown in Fig. 5. As may be seen, the calculated number of moles decreases smoothly with time before reaching equilibrium, without the fluctuations seen in the source data. This behavior indicates essentially no temporal lag between gas temperature (indicated by gas pressure) and the volume wall temperature, even though a temperature difference exists initially. The equilibration times may appear rather long but are probably extended because of the high resolution of the present measurements (± 0.0005 % in number of moles). Similar temperature relaxation due to adiabatic effects has been reported during expansion of gas from a vessel at high pressure into another at lower pressure or vacuum [18]. In this case the difference between measured gas and wall temperatures was found to decrease rapidly to less than 0.010 K after only one-half hour.

The quantities, *p*_0_ *C*_0_/*T*_0_, *p*_1_ *C*_1_/*T*_1_, and *p*_2_ *C*_2_/*T*_2_, for the small, intermediate, and large volumes during the equilibrium time intervals (e.g., Fig. 6) were averaged and their mean values were substituted into Eq. (8) to determine the small reference volume using the previously calibrated value for the large reference volume, i.e., *V_L_* = (939.642 ± 0.022) cm^3^.

**Fig. 7:**
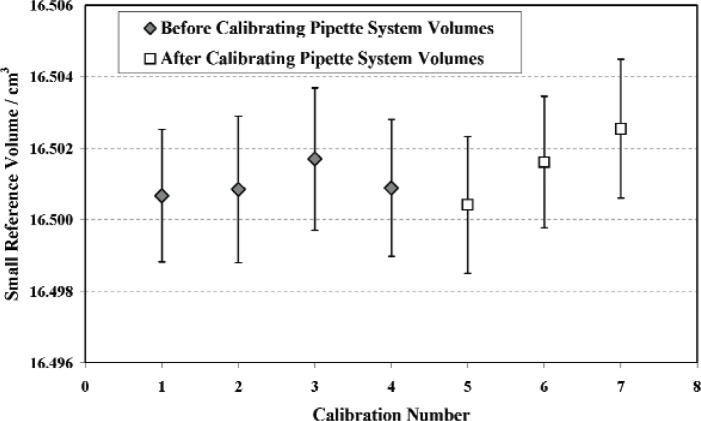
Results of seven repeat calibrations of the small reference volume.

The calibration of the small reference volume was repeated three times before proceeding to the calibration of the volumes in the six pipette systems. After calibrating the other system volumes, the small reference volume was again calibrated three additional times to check its stability in view of possible wear during repeated closing of the four defining valves. If there had been significant wear, the valve stems would penetrate deeper into the reference volume causing its value to decrease. However no evidence of this effect was observed as illustrated by Fig. 7.

The mean value for the small reference volume at 23.0 °C for seven calibrations was 16.5012 cm^3^ with an estimated uncertainty of 0.0021 cm^3^ or 0.013 %. The largest source of uncertainty was from the measurements of temperature (0.01 %), principally from temperature gradients in the small reference volume after the initial fill and from gradients in the large reference volume after the second expansion. The PRTs were mounted at four locations on the small volume and at two locations on the large volume. The next largest contribution to volume uncertainty (0.0064 %) was the result of calibration instabilities of the VCG and the RSGs. Other contributions were due to uncertainties in the large reference volume (0.0023 %) and random scatter among the seven repeat calibrations (0.0034 %). Methods used for estimating the uncertainties are described in Sec. 6.

### 3.3 Reservoir Volumes

#### 3.3.1 Basic Method

The calibration of each reservoir volume followed the same four steps outlined in Sec. 3.2.1. However in this case, it is the volume of the large reservoir that is unknown and its value at 23 °C can be expressed in terms of the known small reference volume at 23 °C, *V_S_* = 16.5012 cm^3^, by rewriting Eq. (8) as

$$V_{L}=\frac{V_{S}[p_{0}C_{S0}/T_{S0}-p_{2}C_{S2}/T_{S2}-(p_{0}C_{S}{}_{0}/T_{S}{}_{0}-p_{1}C_{S}{}_{1}/T_{S1})p_{2}C_{I}{}_{2}T_{I}{}_{1}/p_{1}C_{I}{}_{1}T_{I2}]}{\left(p_{f}C_{Lf}/T_{Lf}\right)}$$ (9)

The intermediate volume between *V_S_* and each reservoir, which must be known in order to measure the volume of the associated pipette (Sec. 3.4) as well as the reservoir fills (Sec. 4), is given by

$$V_{I}=V_{S}(p_{0}C_{S0}/T_{S0}-p_{1}C_{S1}/T_{S1})/(p_{1}C_{I1}/T_{I1})$$ (10)

#### 3.3.2 Measurements

The preparation for and the sequence of measurements in calibrating the reservoir volumes was essentially the same as that already described in Sec. 3.2.2, including the nominal times required for equilibration. As before, the initial starting pressures were nominally 130 kPa and the final pressures after the second expansion were nominally 2000 Pa for the 1000 cm^3^ reservoirs or 700 Pa for the 3000 cm^3^ reservoirs. However there were notable differences which are described below.

After the first expansion into the intermediate volume and allowing time for equilibration, the gas was expanded into the reservoir by slowly opening the pipette entrance valve. This valve remained fully open until thermal equilibration was achieved (12 to 15) h.

The final step involved closing the pipette entrance valve (see Fig. 1) until initial valve seat contact was made. This was done very slowly to minimize the possibility of generating unequal gas pressures between the reservoir and the small reference volume. The valve was then closed with the required torque (14.2 N·m) to reproduce the intermediate volume. After measuring the gas fill pressure and volume temperatures, the valve was reopened by a small amount to re-equalize the pressures in the calibration system and the final step was repeated until a total of ten readings of gas pressure and volume temperatures had been accumulated. The uncertainty in the mean gas pressure measured during multiple closures of the pipette entrance valve was of the order of 0.0001 % (2 times the standard deviation of the mean).

As before the quantities, *p*_0_ *C*_0_/*T*_0_, *p*_1_ *C*_1_/*T*_1_, and *p*_2_ *C*_2_/*T*_2_, for the small, intermediate, and large volumes during the equilibrium time intervals were averaged, and their mean values were substituted into Eqs. (9) and (10) to determine *V_L_* and *V_I_*.

The calibration of the reservoir volume for each pipette system was repeated two times except the system for atmospheric argon, which was repeated only once. A summary of the mean values for the volumes at 23 °C in the calibration system is given in Table A1 of the Appendix. The reservoirs are given labels V039, V040, etc to correspond with labels U039, U040, etc that are stamped into the body of the associated Dörflinger pipettes. The values for reservoir volumes include a small correction for their compressibility corresponding to an anticipated argon fill pressure of ~ 5 Pa. The uncertainties of the reservoir volumes range from 0.018 % to 0.036 % with contributions arising from uncertainties in the small reference volume (0.013 %), the measurement of pressures (0.007 % to 0.01 %), the measurement of volume temperatures (0.01 % to 0.03 %), and scatter among repeated calibrations (0.003 % to 0.02 %). A discussion of uncertainty estimates is given in Sec. 6.

### 3.4 The Dörflinger Pipette Volumes

#### 3.4.1 Basic Method

The calibration of each pipette followed essentially four steps. (1) The associated reservoir was filled with nitrogen gas and, after sufficient time for equilibration, the fill pressure (*p_f_*) and reservoir temperature (*T_Lf_*) were measured. (2) The first aliquot of gas was drawn into the pipette and then expanded into the small reference volume and interconnecting intermediate volume, all of which had been previously evacuated to base pressure. (3) After equilibration, the pressure of the expanded gas from the aliquot and the temperatures of the volumes were measured and, from these data, the volume of the Dörflinger pipette was calculated using Eq. (13) given below. (4) The second and third steps were repeated for subsequent aliquots drawn from the reservoir.

The number of moles of gas remaining in the reservoir after removing the *i*-th aliquot may be derived on the basis of conservation of mass as

$$n_{Li}=\frac{n_{Lf}}{{\left(1+r_{V}\right)}^{i}}$$ (11)

and the number of moles in the *i*-th aliquot is given by

$$n_{Di}=r_{V}n_{Li}=\frac{r_{\nu }n_{Lf}}{{\left(1+r_{V}\right)}^{i}}$$ (12)

where *n_L f_* = *p_f_ V_L_ C_L f_*/(*R T_L f_*) is the initial number of moles in the reservoir and *r_V_* is the pipette to reservoir volume ratio defined by Eq. (6). This derivation is based on the additional assumption that the pressure and temperature of the gas in the pipette and reservoir are the same at the time of initial contact of the pipette entrance valve seat.

By assuming the number of moles in the *i*-th aliquot is conserved after expansion, the pipette to reservoir volume ratio may be expressed as

$$r_{V}\frac{[V_{I}(p_{i}C_{Ii}/T_{Ii})+V_{S}(p_{i}C_{Si}/T_{Si})]{\left(1+r_{V}\right)}^{i}}{V_{L}(p_{f}C_{Lf}/T_{Lf})}$$ (13)

where *p_i_* is the pressure in the system after expansion of the *i*-th aliquot. In deriving this equation recall that the pipette volume is included as part of the intermediate volume (see Sec. 2.4.4). The volume ratio is obtained by an iterative solution of Eq. (13). The Dörflinger pipette volume *ν_D_*′ is then determined from Eq. (6) using the calibrated value of the reservoir volume, *V_L_*, and the appropriate value of the factor *f* (1.000089 for the ^38^Ar systems, 1.000037 for the artificial mixture systems).

#### 3.4.2 Measurements

With the system initially at base pressure, the selected pipette/reservoir system, the intermediate volume, and small reference volume were filled with nitrogen gas to a nominal pressure of 130 kPa and allowed to equilibrate, typically for 15 hours. Ten sets of readings of fill pressure and reservoir temperature were taken, each time after closing the pipette entrance valve slowly to minimize creating pressure differences between the reservoir and the small reference volume.

**Fig. 8:**
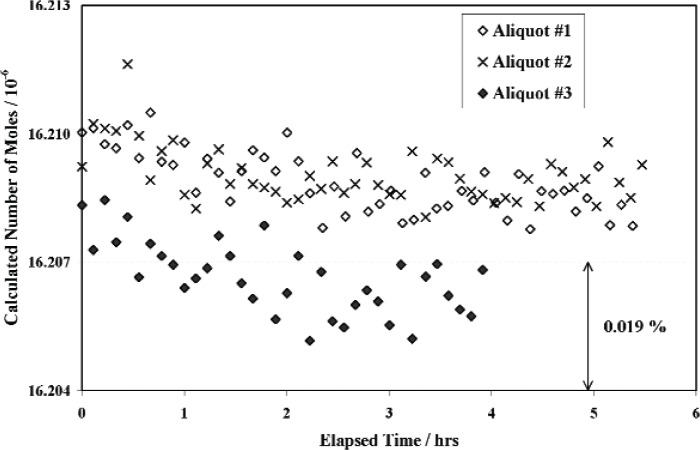
Calculated number of moles in three successive aliquots of nitrogen drawn into the pipette from the reservoir and expanded into the intermediate and small reference volumes.

With the pipette entrance valve closed to isolate the filled reservoir, the intermediate and small reference volumes and the pipette were evacuated to base pressure. The pipette exit valve was then closed with the required torque (14.2 N·m) and the entrance valve was opened allowing nitrogen to flow into the pipette. After a short equilibration time (a few minutes)^6^, the entrance valve was again closed slowly before applying the required closing torque. The exit valve was then opened to expand the aliquot of nitrogen into the intermediate and small reference volumes. After equilibration for 1 to 2 hours (see Fig. 8), several sets of pressure and temperature readings were taken. The volume ratio was calculated from Eq. (13) using mean values of the quantities, *p_i_ C_Si_*/*T_Si_*, *p_i_ C_Ii_*/*T_Ii_*, and *pf C_Lf_*/*T_Lf_*, and the previously calibrated values for the small reference, intermediate and reservoir volumes. The pipette volume was then calculated from Eq. (6). Nominal pressures in the small reference volume after expansion were 225 Pa for pipettes U039 and U040, 280 Pa for U041 and U043 and 95 Pa for U042 and U046. The differences were due to different pipette volumes and intermediate volumes (see Fig. 3).

The value for the volume of each pipette was determined as the mean of values determined from successive expansions of three aliquots. A second reservoir fill was used to provide additional volume measurements based on the expansion of another three aliquots for pipettes U039 and U040 in the ^38^Ar systems but no systematic difference in results from the two fills was observed. In principle, the number of moles in each successive aliquot should be reduced by the pipette/reservoir volume ratio (nominally 1/10^4^). However, as may be seen in Fig. 8, there is some scatter due to non-reproducibility of closing the pipette entrance and exit valves, particularly the exit valve. Based on these results, the resolution of drawing a single aliquot from the reservoir was estimated as two times the standard deviation of a single measurement of pipette volume about the mean value. This was found to be not larger than 0.025 %, which is the resolution in pipette volume associated with the resolution of the torque wrench (Sec. 2.4.4).

A summary of mean values for pipette volumes at 23 °C are given in Table A2 in the Appendix along with nominal values stated by the manufacturer. As may be seen, the nominal stated values are systematically higher by amounts ranging from 0.7 % to 1.4 % indicating that a lower closing torque may have been used by the manufacturer to define the pipette volume. The combined uncertainties in the mean values are 0.033 % for U39, U40 and range from 0.078 % to 0.89 % for similar sized U42, U46, and from 0.046 % to 0.053 % for the larger U41, U43. The large variations in uncertainties reflect differences not only in pipette size but also in the associated intermediate volumes. As a result, final pressures ranging between 90 Pa and 290 Pa gave rise to contributions from the measurement of pressure in the range 0.02 % to 0.06 %. Other contributions were from uncertainties in the small reference volume (0.002 % to 0.004 %), the intermediate volume (0.015 % to 0.03 %), the measurement of volume temperatures (0.01 % to 0.05 %), and uncertainty in the mean value due to scatter among repeated calibrations of pipette volume (0.01 % to 0.015 %).

## 4. Measurement of Requisite Quantities of Argon

### 4.1 Pipette Systems for ^38^Ar

#### 4.1.1 Basic Fill Method

The filling of reservoirs with the requisite molar quantities of ^38^Ar involved essentially three steps. First, the small reference volume was filled with ^38^Ar and, after sufficient time for equilibration, the initial gas pressure (*p*_0_) and volume temperature (*T_S_*_0_) were measured allowing the system molar quantity (*n_S_*_0_) to be calculated from Eq. (2). Second, the argon gas was expanded into the intermediate and reservoir volumes and, after equilibration, the pressure of the expanded gas (i.e., the reservoir fill pressure *p_f_*) was calculated from Eq. (1) using *n_S_*_0_ and previously calibrated values for the reference, intermediate, and reservoir volumes and their equilibrium temperatures:

$$p_{f}=\frac{n_{S0}}{(V_{S}C_{Sf}/RT_{Sf}+V_{I}C_{If}/RT_{If}+V_{L}C_{Lf}/RT_{Lf}}$$ (14)

Finally the molar quantity of argon in the reservoir was determined from the calculated fill pressure *p_f_* :

$$n_{Lf}=p_{f}(V_{L}C_{Lf}/RT_{Lf})$$ (15)

#### 4.1.2 Measurements

Because of the rather low fill pressures (≈ 3 Pa to 10 Pa), the reservoirs were evacuated to the lowest possible base pressures (3 × 10^−6^ Pa to 5 × 10^−6^ Pa) prior to filling, ensuring a negligible contribution from residual gases to the molar quantities of argon in each reservoir. The requirements for the small reference volume and the argon gas admission plumbing were less stringent due to much higher initial pressures (≈ 230 Pa to 1900 Pa) during argon gas fills and so their starting base pressures were somewhat higher (1 × 10^−5^ Pa to 3 × 10^−5^ Pa).

The addition of ^38^Ar gas into the small reference volume was accomplished in two stages. First the seal on the glass vial containing the ^38^Ar was broken, releasing the gas into the gas admission plumbing, which was initially isolated from the small reference volume and the TM pump. The ^38^Ar was then admitted very slowly to the reference volume while adding a compensating amount of nitrogen gas to the CDG low-pressure or reference side to avoid CDG over-ranging that would cause unwanted zero shifts. After isolating the ^38^Ar in the reference volume, the gas was allowed to equilibrate until the calculated number of moles of ^38^Ar, based on readings of *p*_0_ and *T_S_*_0_ became essentially independent of time (similar to the behavior shown in Fig. 6). A mean value for the initial number of moles in the reference volume (*n_S_*_0_) was calculated based on data taken after equilibrium was established.

**Fig. 9:**
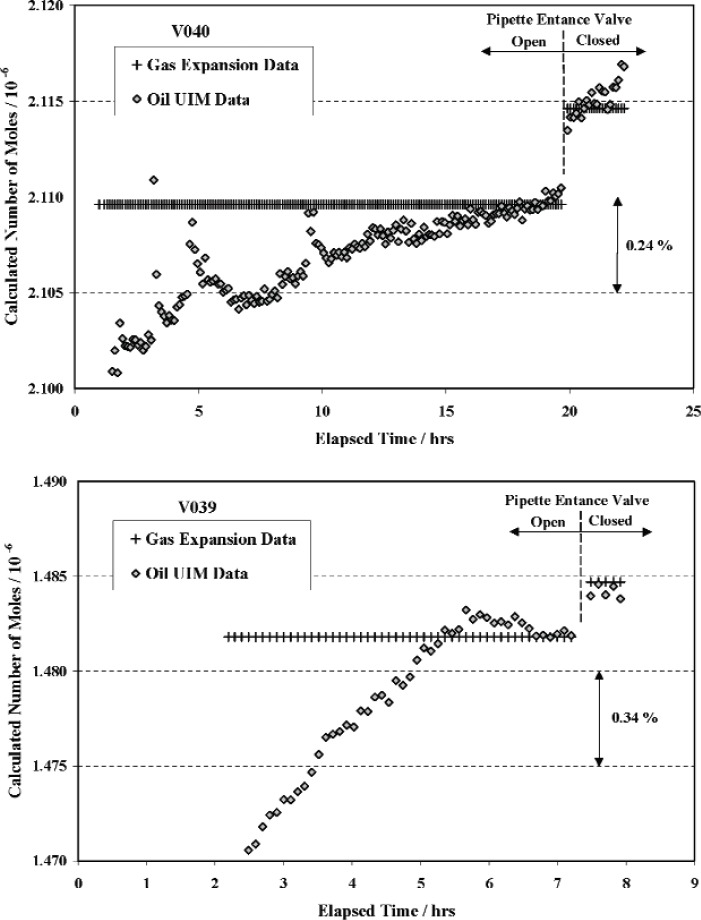
Calculated number of moles in reservoir V040 (upper) or reservoir V039 (lower) as a function of time where the fill pressure is determined by a gas expansion method (+ symbols) or by direct measurement using a 140 Pa Oil UIM (diamond symbols).

With the pipette entrance valve fully open, the ^38^Ar was slowly expanded into the intermediate plumbing and the reservoir, again taking care to minimize large excursions in the CDG readings by removing compensating amounts of nitrogen from the CDG reference side. Figure 9 shows the calculated number of moles in reservoirs V040 and V039 as a function of time after expansion. The + symbols refer to the calculated molar quantity of argon based on a calculated pressure *p_f_* given by Eq. (15). The pipette entrance valve, when fully open, adds a nominal volume of 2 cm^3^ to the intermediate volume which, when included, results in a lower calculated values for fill pressure and hence molar quantity of ^38^Ar. The diamond symbols refer to results obtained by direct measurement of fill pressure using the oil UIM, which were used primarily to determine onset of thermal equilibrium between the gas and volume walls. As mentioned earlier and discussed below, the accuracy of directly measuring fill pressures of 10 Pa or less is severely limited by zero instability of the CDG.

As before, thermal equilibrium was assumed to occur when the calculated number of moles in the system ceased to change with time. After thermal equilibrium was achieved, the entrance valve was closed slowly until initial contact of the valve seat was made before closing with the required torque (14.2 N·m). Readings of temperature of the reference, intermediate, and reservoir volumes were taken and the final fill pressure and molar quantity of ^38^Ar in the sealed reservoir were calculated via Eqs. (14) and (15), respectively. The entrance valve was then opened a small amount to allow pressure in the reservoir and the system to re-equalize and the above closing procedure was repeated for several readings.

The mean molar quantity of ^38^Ar (from Batch 2.8.3.21) in reservoir V039 was calculated as 1.4847 × 10^−6^ moles with a combined uncertainty of 0.036 %. The mean value for the molar quantity of ^38^Ar (from a combination^7^ of Batch 2.8.3.24 and Batch 2.8.3.25) in reservoir V040 was calculated as 2.1146 × 10^−6^ moles with a combined uncertainty of 0.029 %. The major contributions to the combined uncertainties were due to uncertainties in the small reference volume (0.013 %), impurities in the source gas (0.01 % maximum), the measurement of initial fill pressures (0.019 % to 0.026 %), and the measurement of volume temperatures (0.01 % to 0.015 %). The final results for molar quantities in the two pipette systems are summarized in Table 1 of Sec. 5.

Because of the rather low fill pressures (≈ 5.71 Pa for V040 and ≈ 3.86 Pa for V039), the calculated molar quantity based on measured fill pressures is very sensitive to zero instabilities of the CDG. The zero-pressure offsets of the CDG were measured after filling each reservoir and found to be +0.022 Pa for V040 and −0.013 Pa for V039. Corrections for these zero offsets (nominally 0.4 % of the respective fill pressures) were applied to all direct measurements of fill pressure even though it was not possible to know their earlier history. While reasonably accurate for the very last fill pressure measurements, the corrections rapidly become less reliable for earlier measurements. Differences in zero drift of the CDG during the molar fills of V040 and V039 may explain the apparent large difference in time required achieving equilibrium as seen in Fig. 9. The “spikes” in the results for V040 were artifacts generated by adjustments to the reference side pressure in order to restore the CDG reading to near zero.

The molar quantities based on direct fill pressure measurements, while significantly less accurate, provide a check on the results obtained above by gas expansion methods. The direct pressure measurements yield mean values for molar fills of V040 and V039 that are 0.023 % higher and 0.036 % lower than respective molar quantities obtained by expansion methods, which have estimated uncertainties of 0.029 % and 0.036 %. The relatively small differences of the direct measurement results from the expansion results may be fortuitous since they correspond to an error in zero offset of only 0.001 Pa, which is essentially the pressure resolution of the CDG.

### 4.2 Pipette Systems for Artificial Mixtures of ^40^Ar and ^36^Ar

#### 4.2.1 Basic Preparation Method

In filling reservoirs with artificial mixtures, the first step of the method outlined in Section 4.1.1 was subdivided.

(1a) The small reference volume was filled with the major component (^40^Ar) and, after sufficient time for equilibration, the pressure of the major component (*p_M_*) and the reference volume temperature were measured.
(1b) The minor component (^36^Ar) was then added to the small reference volume and, after additional time for equilibration, the total gas pressure *p*_0_ and the volume temperature were measured to determine the molar ratio for the artificial mixtures:
  $$\frac{n_{S40}}{n_{S36}}=\frac{n_{S40}}{n_{S0}-n_{S40}}=\frac{p_{M}C_{SM}/T_{SM}}{p_{0}C_{S0}/T_{S0}-p_{M}C_{SM}/T_{SM}}$$ (16)
  where *n_S_*_36_ and *n_S_*_40_ are the number of moles of ^36^Ar and ^40^Ar, respectively, in the small reference volume, *n_S_*_0_ is the total number of moles of the artificial mixture, *p_M_* is the partial pressure of ^40^Ar and *p*_0_ is the total pressure of the mixture. To the extent that temperature of the small reference volume does not change during the preparation of the mixture, the molar ratio is given by the ratio of their partial pressures.

The second and third steps of Sec. 4.1.1 were then followed to determine the molar quantity of artificial mixture in a given reservoir.

#### 4.2.2 Measurements

In the preparation of the artificial mixtures it was important that, when adding the minor component (^36^Ar) to the major component (^40^Ar) in the reference volume, the starting pressure of the minor component in the gas admission plumbing was sufficiently high to minimize back-streaming of the major component. Therefore, an auxiliary CDG (not shown in Fig. 3) was connected to the gas admission plumbing to read the starting pressures.

**Fig. 10:**
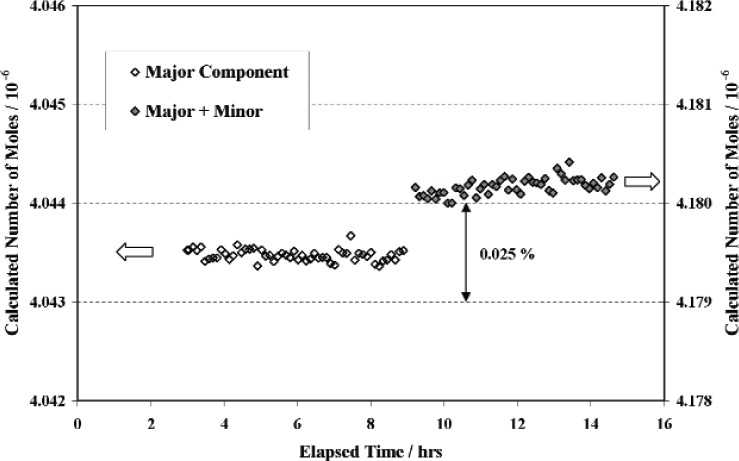
Data obtained during preparation of the nominal 30:1 mixture of ^40^Ar:^36^Ar.

The gas admission plumbing was first filled with ^40^Ar to a starting pressure nominally 3 to 5 times larger than the final desired gas pressure *p_M_*. The ^40^Ar was then admitted slowly to the small reference volume until pressure *p_M_* was achieved (327 Pa, 602 Pa, and 1890 Pa for the 1:1, 30:1, and 300:1 systems, respectively). After equilibration for a few hours, several readings of *p_M_* and *T_SM_* were taken and averaged to determine *n_S_*_40_. The gas admission plumbing was then evacuated to base pressure before filling with ^36^Ar to a starting pressure nominally an order of magnitude larger than the desired total gas pressure *p*_0_. As before, the ^36^Ar was admitted slowly to the small reference volume until the desired pressure *p*_0_ was achieved (652 Pa, 622 Pa, and 1897 Pa for the 1:1, 30:1, and 300:1 systems^8^, respectively). After a short equilibration interval of 10 to 20 minutes, several readings of *p*_0_ and *T_S_*_0_ were taken and averaged to determine *n_S_*_0_. The values for *n_S_*_0_ and *n_S_*_40_ were then used to determine molar ratios for the three artificial mixtures via Eq. (16). Typical data taken during preparation of the mixtures is shown in Fig. 10.

The molar ratios for the ^40^Ar:^36^Ar mixtures were determined to be 0.9974, 29.69, and 285.7 with combined uncertainties of 0.06 %, 0.06 %, and 0.08 %, respectively. The major contributions to the combined uncertainties were due to uncertainties arising from impurities in the source gases (0.022 % or less), measurements of pressure (0.021 % to 0.006 %), and random scatter in measurements of the mean molar quantities (0.002 % to 0.061 %).

The filling of reservoirs with requisite molar quantities of artificial mixtures followed the same methodology already described in Sec. 4.1.2. The molar quantities of ^40^Ar:^36^Ar mixtures with nominal ratios 1:1, 30:1, and 300:1 were determined to be 3.8008 × 10^−6^ moles, 3.9908 × 10^−6^ moles, and 12.1503 × 10^−6^ moles with combined uncertainties of 0.026 %, 0.025 %, and 0.022 %, respectively. Major contributions to the combined uncertainties were due to uncertainties arising from impurities in the source gases (0.01 % or less), uncertainties in the small reference volume (0.013 %), the measurement of pressures (0.006 % to 0.011 %), and the measurement of temperatures (0.010 % to 0.014 %).

The results for the molar ratios and molar quantities of the mixtures in the reservoirs are summarized in Table 1 of Sec. 5.

### 4.3 Preparation of Glass Ampoules Containing ^40^Ar / ^36^Ar Mixtures

Although not mentioned earlier, the calibration system also included three glass storage reservoirs that were evacuated and subjected to high-temperature baking at the same time as the metal components of the system. Each glass reservoir consisted of fifteen closed glass tubes (3.8 mm internal diameter) nominally 160 cm long that were connected to a hollow glass base, which had a metal-bellows inlet valve. Each glass reservoir was used to store as much of the excess artificial mixture as possible after the associated reservoir had been filled and sealed.

After all volume and molar fill calibrations were completed, the glass reservoirs were removed from the calibration system and the glass tubes were flame-tipped into ampoules nominally 10 cm long resulting in approximately 150 ampoules for each artificial mixture. The molar quantity in each ampoule was estimated to be nominally 2.2 × 10^−10^ moles for the ^40^Ar:^36^Ar mixture with molar ratio 29.69 and nominally 6.5 × 10^−10^ moles for mixtures with molar ratios 0.9974 and 285.7.

As a minimum, these ampoules with their precisely known molar ratios will be a valuable resource for laboratories involved in geochronological studies. If there is sufficient demand, these ampoules could be distributed by NIST as sets of three Standard Reference Materials (SRMs).

### 4.4 Pipette System for Atmospheric Argon

The source gas was obtained by filling an evacuated steel reservoir with outdoor air rather than laboratory air because of possible contamination with artificially introduced argon. The outdoor air was passed through a liquid nitrogen trap to remove any moisture as it was introduced it into the calibration system^9^.

The same measurement procedures described earlier in Sec. 4.1.2 were used to fill reservoir V043 with air containing atmospheric argon. The molar quantity of the air in the reservoir was determined to be 4.0433 × 10^−6^ moles with an uncertainty of 0.023 %.

## 5. Summary of Final Results for the Pipette Systems

The final results for the preparation of six pipette systems are given in Table 1 where *n_Lf_* is the initial^10^ molar quantity of ^38^Ar and of the artificial mixtures of ^40^Ar:^36^Ar in their respective reservoirs and *n_D_*_1_ is the molar quantity in the first aliquot to be delivered by the Dörflinger pipette. Also given are the pipette/reservoir volume ratios of the pipette systems and the molar ratios of the artificial mixtures along with estimated combined uncertainties.

The key equation at the time of use by the USGS is Eq. (12), which gives the number moles in the *i*-th aliquot:

$$n_{Di}=r_{V}n_{Li}=\frac{r_{V}n_{Lf}}{{\left(1+r_{V}\right)}^{i}}$$ “(12)”

The uncertainty contributions to *n_Di_* that arise from uncertainties in quantities determined at the time of system calibrations, such as *r_V_* and *n_Lf_*, can be readily derived from the above equation. However uncertainty contributions that arise at the time of use will be more difficult to evaluate and yet these can have a significant effect on the uncertainty of the molar quantity in the aliquots. There will be two primary sources for such uncertainties. The first source is the limited reproducibility of closing the entrance and exit valves of the pipette. The second source is if there is a difference in temperature of the gas in the pipette from that of the gas in the reservoir. Taking these potential sources into account, the combined relative uncertainty in *n_Di_* may be estimated from

$${\left(\frac{u(n_{Di})}{n_{Di}}\right)}^{2}={\left(\frac{u(n_{Lf})}{n_{Lf}}\right)}^{2}+F_{i}^{2}{\left(\frac{u(r_{V})}{r_{V}}\right)}^{2}+{\left(\frac{\Delta n}{n_{D}}\right)}^{2}+{\left(\frac{\Delta T}{T_{D}}\right)}^{2}$$ (17)

where the factor *F_i_* = [1 − *i r_V_*/(1 + *r_V_*)], and the first and second terms are the relative uncertainties in *n_Lf_* and *r_V_* as given in columns four and six of Table 1, respectively. The third term is the relative uncertainty in molar content due to non-reproducibility of closing the entrance and exit valves of the pipette and ∆*T* = *T_L_* − *T_D_* is the difference in absolute temperature of the gas in the reservoir and the pipette. The *ad hoc* addition of the third and fourth terms assumes that valve closure variations and temperature differences are normally distributed.

A reasonable estimate for the third term in Eq. (17) may be given by the resolution of drawing a single aliquot from the reservoir. In Sec. 3.4.2 this was taken as two times the standard deviation of a single measurement of pipette volume about the mean and was found to be 0.025 %. This is consistent with the resolution of the torque wrench used to define the pipette volume (see Sec. 2.4.4). The fourth term can be made negligible by controlling the temperature difference between the reservoir and pipette to 10 mK or less. The average temperature difference during the present calibrations was generally 0.005 K or less, but these measurements were carried out under highly controlled conditions.

Table 1 also presents the molar content (*n_D_*_1_) of the first aliquot as calculated from Eq. (12) and its relative uncertainty as calculated from Eq. (17) for a negligible temperature difference between pipette and reservoir. The relative uncertainty of the aliquot content is rather insensitive to the number of aliquots previously drawn. For example, it remains essentially constant even after 1000 aliquots at which point the molar content of the aliquot and the reservoir will be smaller by 10 %.

It will be important for the users of the ^38^Ar pipette systems to monitor the pipette and reservoir temperatures and, if necessary, include the difference in their estimate of uncertainty. It will be especially critical to close the exit valve with the torque wrench used in the present calibrations (and supplied with the pipettes) and to use the same torque reading, namely, 14.2 N·m (126 lbf·in). Differences among torque wrenches can readily exceed 1 % of reading. A 1 % error would correspond to an additional uncertainty of 0.06 % in volume and molar content of the pipette and would have to be included as another term in the uncertainty estimate given by Eq. (17). Periodic calibrations of the torque wrench are recommended to establish the stability of its reading over time.

## 6. Estimate of Uncertainties

Evaluations of uncertainty in the measured quantities (measurands) were carried out following guidelines given in Ref. [21]. There were two types of measurands in the present calibrations, namely, volume temperature (surrogate for gas temperature) and gas pressure. Uncertainties in the measurands arising from random effects were evaluated by statistical analysis (Type A evaluations) and found to be negligibly small. However, the Type A uncertainties due to scatter in repeated calibrations of the large reference volume and of molar quantities in the artificial mixtures were non-negligible but generally smaller than the calibration uncertainties arising from systematic effects in the measurands. These uncertainties were evaluated by non-statistical methods (Type B evaluations) as described below.

The volume temperature was calculated as the mean of PRT readings associated with a particular volume (2 to 7 PRTs). The uncertainty in volume temperature was primarily due to temperature gradients within the volume, calibration uncertainties of the PRTs, and the calibration uncertainty of the Digital Multimeter (DMM) used to measure PRT resistances. The uncertainty due to temperature gradients was modeled by a normal distribution such that there was a 2 out 3 chance that the volume temperature was in the interval between the maximum and minimum PRT readings. In this case the uncertainty due to this source of error was estimated as one-half the difference between the maximum and minimum PRT readings multiplied by a coverage factor of *k* = 2. In general, the uncertainty due to temperature gradients was about an order of magnitude larger than that due to PRT calibration uncertainties. The calibration uncertainty of the DMM contributed a maximum uncertainty of 0.036 K, which appears as a common error for all PRTs. The latter uncertainty will contribute to uncertainties in the calibration of molar quantities but not to volume calibration uncertainties since volume temperatures appear in both numerator and denominator of the defining equations.

The uncertainty in gas pressure was primarily due to three sources, uncertainty in the calibration equation used to convert readings of the high-accuracy transducers (VCG or 3 RSGs) into “true” pressures, long-term shifts in calibration of the transducers, and zero instabilities of the CDG. The long-term instabilities of the transducers were evaluated on the basis of three calibrations performed against a mercury UIM primary pressure standard at NIST during the course of the present work. The uncertainty due to calibration shifts was modeled by a normal distribution such that there was a 2 out of 3 chance that the transducer response at a given pressure was in the interval between maximum and minimum transducer readings for the three calibrations. The uncertainty due to calibration instability was then estimated as one-half the difference between the maximum and minimum transducer readings multiplied by a coverage factor of *k* = 2. The uncertainty due to CDG zero instability was estimated as ± 0.015 Pa based on observed drifts in the CDG zero pressure reading during time intervals comparable to those required for thermal equilibration.

The use of spreadsheets greatly facilitated not only the calculation of a desired measurement result (e.g., reservoir or reference volume, molar quantity, etc.) but also the contribution to the uncertainty of the result arising from estimated uncertainties in the measurands. Each uncertainty contribution was evaluated as the change in measurement result that is produced when the associated measurand is changed by its uncertainty, while taking into account possible correlation effects among the measurands. The combined uncertainty of the measurement result was then obtained as the root-sum-of-squares of the uncertainty contributions from the measurands.

As noted earlier^2^, the term uncertainty in this report refers to expanded uncertainty with a coverage factor *k* = 2. This coverage factor is likely to approximate a 95 % level of confidence in the present work for the following reasons. Although degrees of freedom of Type A uncertainties are easily determined using statistical methods, the evaluation of degrees of freedom associated with Type B uncertainties is more problematic. If Type B uncertainties are evaluated in such a way that the probability of the measurand lying outside the uncertainty limits is extremely small (e.g., a rectangular probability distribution) then the degrees of freedom become infinitely large. Such an approximation, which cannot be fully justified in the present work, is not necessarily unrealistic since the Type B evaluations were carried out in a manner that attempted to avoid an underestimation of the uncertainties. Since the Type B uncertainties in the present work were generally dominant, the “effective degrees of freedom” [21] of the combined (Type A and Type B) uncertainties in the results are expected to be quite large. For example, the 95 % confidence limits for degrees of freedom in the range 30 to ∞ correspond to a coverage factor in the range 2.04 to 1.96, respectively.

## 7. Discussion of Results

In filling the reservoirs with requisite molar quantities, it was implicitly assumed all of the argon that expanded into the reservoir remained in its gaseous phase and that only a negligible amount was adsorbed on the interior walls. However at low fill pressures that range from 3.9 Pa to 5.7 Pa for the ^38^Ar systems and from 3.2 Pa to 10.1 Pa for the ^40^Ar:^36^Ar systems, gas behavior is dominated by gas-surface collisions and so the issue of gas adsorption needs to be addressed.

Theoretical isotherms for physical adsorption have been developed and fitted to experimental data for a variety of gases, including argon, over a wide range of adsorbing surfaces, temperatures, and pressures [25]. By interpolating between isotherms fitted to argon data at 125 K and 500 K, we estimate the coverage (fraction of a monolayer adsorbed on the surface) at room temperature and at pressures in the range 5 Pa to 10 Pa was less than or equal to 10^−5^.

The number of moles adsorbed to the interior surface of a reservoir, *n_ad_*, may be expressed as

$$n_{ad}=\frac{\Theta \times N_{S}\times S_{area}}{N_{A}}$$ (18)

where Θ ≤ 10^−5^ is the coverage, *N_S_* = 8.54 × 10^18^ m^−2^ is the number of argon molecules per unit surface area in a monolayer [26], *S_area_* is the interior surface area of the reservoir, and *N_A_* = 6.022 × 10^23^ mol^−1^ is the Avogadro constant [6]. The 1000 cm^3^ and 3000 cm^3^ reservoirs have internal surface areas that are nominally 0.05 m^2^ and 0.115 m^2^ and so Eq. (18) predicts the number of moles adsorbed to be less than or equal to 7.1 × 10^−12^ and 1.63 × 10^−11^, respectively. Given the molar quantities of Ar in the reservoirs (see Table 1), the fraction of gas that will be in the adsorbed phase will not be larger than 5 × 10^−6^ or 0.0005 % for reservoirs of either size, which is negligible when compared to estimated uncertainties in their molar contents. This conclusion is supported by the agreement between molar quantities determined by gas expansion methods and those determined by direct measurement of the fill pressures (Sec. 4.1.2).

The pipette/reservoir systems are expected to have a relatively long “shelf life” in the absence of unintended leaks to or from the reservoirs due to improper valve operation. Even after drawing 1000 aliquots from a reservoir, the molar quantity remaining is reduced by only 10 %. However there are two effects that could compromise molar quantity. For systems containing artificial mixtures, the issue would be a leak of atmospheric ^40^Ar into the reservoir. Based on leak tests performed on the pipette systems (see Secs. 2.2 and 2.4.2), the accumulation of atmospheric ^40^Ar is estimated to be less than 3 × 10^−14^ moles per year, which is a negligible fraction of the ^40^Ar in the reservoir (≈ 10^−5^) even after many decades. For the ^38^Ar reservoirs the most significant issue is outgassing of hydrogen from their interior walls. As mentioned in Sec. 2.2, the reservoirs were pretreated by vacuum firing in order to reduce hydrogen outgassing. Outgassing rates routinely observed at room temperature for hydrogen after vacuum firing are nominally 10^−14^ Torr liter s^−1^ cm^−2^ [27]. This corresponds to a hydrogen accumulation rate in the 1000 cm^3^ and 3000 cm^3^ reservoirs of about 8.5 × 10^−9^ mole per year and 1.95 × 10^−8^ mole per year, respectively, or a 0.5 % increase in molar quantity per year due to the hydrogen outgassing. The molecular weight of hydrogen is far removed from that of argon and so the accumulation of hydrogen should not interfere significantly with mass spectrometry centered on argon, at least for the immediate future. If necessary, the hydrogen could be removed by chemical means after an aliquot is drawn from the reservoir. In any case, the accumulation of hydrogen would not affect the quantity of argon removed with each aliquot since it is the pipette to reservoir volume ratio that determines the fractional amount removed.

## 8. Concluding Remarks

NIST has developed argon isotope reference standards in the form of pipette systems that will deliver small aliquots of high purity argon with exceptionally low uncertainty. Two of the pipette systems will deliver small aliquots of ^38^Ar (nominally 2 × 10^−10^ moles) with an uncertainty of 0.06 %, which is approximately a factor of four better than the target levels of 0.25 % stated in the project objective. This level of uncertainty should be achievable if the user exercises sufficient care when closing the pipette entrance/exit valves and minimizes the pipette/reservoir temperature differences. Three other pipette systems will deliver aliquots (nominally 4 × 10^−10^ moles) of ^40^Ar:^36^Ar mixtures with similar uncertainty and with molar ratios of 0.9974 ± 0.06 %, 29.69 ± 0.06 %, and 285.7 ± 0.08 %. The artificial mixtures will be used by USGS to calibrate the mass discrimination of their mass spectrometers, before using the ^38^Ar pipette to measure the concentration of the radiogenic argon (^40^Ar^*^) in MMhb-2. The USGS will also re-determine the isotopic composition of atmospheric Ar in order to improve the accuracy of correcting for atmospheric argon in MMhb-2 (see Appendix A1).

Upon completion of the USGS calibrations, the MMhb-2 mineral standard will be certified by NIST for its K and Ar concentrations and distributed as a Standard Reference Material (SRM). The new SRM and the NIST-calibrated transportable pipette systems have the potential for dramatically improving the accuracy of interlaboratory calibrations and thereby the measured ages of geological materials, by as much as a factor of ten.

Ampoules of the three ^40^Ar:^36^Ar mixtures (nominally 5 × 10^−10^ moles in each) have been prepared as a byproduct of the present work and, depending on interest, may be distributed by NIST as a set of three SRMs.

## Figures and Tables

**Table 1:** Summary of final results for the six pipette systems. The combined uncertainties were estimated with a coverage factor *k* = 2.

System	Identifiers	Initial molar quantity	Combined uncertainty				
	Reservoir	*n_Lf_*	(%)	Volume ratio *r_V_* (10^−4^)	Combined uncertainty^a^ (%)	Molar ratio ^40^Ar/^36^Ar	Combined uncertainty (%)

	Pipette	*n_D_*_1_	(%)				
^38^Ar	V039	1.4847E-06	0.036	1.0554	0.037		

	U039	1.567E-10	0.058				

^38^Ar	V040	2.1146E-06	0.029	1.0937	0.038		

	U040	2.313E-10	0.054				

^40^Ar:^36^Ar 1:1	V042	3.8008E-06	0.026	1.0585	0.096	0.9974	0.06

	U042	4.023E-10	0.10				

^40^Ar:^36^Ar 30:1	V041	3.9908E-06	0.025	0.9834	0.058	29.69	0.06

	U041	3.924E-10	0.07				

^40^Ar:^36^Ar 300:1	V046	1.2150E-05	0.022	0.3320	0.084	285.7	0.08

	U046	3.997E-10	0.09				

Atmospheric Ar	V043	4.0429E-06	0.024	0.9778	0.052		
				
	U043	3.953E-10	0.06				

a Equal to the root-sum-square of the uncertainties in ν*_D_* and *V_L_*, which are given in column six of Table A2 and column five of Table A1.

## Footnotes

1 A brief description of argon dating techniques is given in Appendix A1.

2 Unless noted otherwise, the term uncertainty in this report refers to expanded uncertainty, which is given by the product of the standard uncertainty and a coverage factor *k* = 2. The value *k* = 2 is used at NIST for calculating expanded uncertainty, in keeping with current international practice (see Ref. [21]).

3 The unit of pressure, the pascal (Pa), is defined as a Newton per square meter (N/m^2^).

4 Certain commercial equipment, instruments, or materials are identified in this paper to foster understanding. Such identification does not imply recommendation or endorsement, nor does it imply that the equipment identified are necessarily the best available for the purpose.

5 The equation of state of *real* gases may be represented by *z* = *pV*_M_/*RT* = 1 + *B_V_*/*V*_M_ + *C_V_*/*V*_M_^2^ + *D_V_*/*V*_M_^3^ +…. where *V*_M_ is the molar volume, *B_V_*, *C_V_*, and *D_V_* are respectively termed the second, third, and fourth virial coefficients. The maximum pressure (130 kPa) in the present work was sufficiently low, requiring only the second virial term in the series expansion to accurately represent the equation of state.

6 When filling a pipette from its reservoir, the volume expansion of the gas is only one part in 10 000 resulting in minimal adiabatic cooling and therefore minimal thermal equilibration time.

7 The molar quantity of ^38^Ar in either batch alone would have provided an aliquot size of only ≈ 1 × 10^−10^ moles, which is 50 % of the smallest desired aliquot size (nominally 2 × 10^−10^ moles).

8 The 300:1 system utilized a combination of a 3000 cm^3^ reservoir with a 0.1 cm^3^ pipette, which enabled the use of a higher fill pressure and thus a higher partial pressure of the minor component for improved accuracy of the molar ratio.

9 In hindsight, it would have been preferable to remove moisture by passing the air through a desiccant because of the (small) risk of argon isotopic fractionation due to the somewhat higher condensation point of argon (87.3 K) relative to nitrogen (77.3 K).

10 From the perspective of the pipette systems users.

## Appendix

### A1. Brief Description of ^40^K / ^40^Ar and ^40^Ar / ^39^Ar Dating Methods

The K-Ar dating method is based on ^40^K, a radioactive isotope of potassium that dual decays to ^40^Ca (beta emission) and ^40^Ar (electron capture) with a half-life of 1250 million years. The half-life is such that measurable quantities of radiogenic argon (^40^Ar^*^) will have accumulated in potassium-bearing minerals of nearly all ages. With current techniques it is possible to measure the age of igneous rocks as young as a few thousand years as well as oldest rocks known [1]. The decay to ^40^Ca is not as useful as a dating tool because ^40^Ca is the most abundant of the calcium isotopes and is so ubiquitous that it is difficult to distinguish the radiogenic calcium from the calcium present in the minerals and rocks at the time of their formation. However, some specific minerals are amenable to K-Ca dating.

In the conventional K-Ar dating method, the potassium concentration is measured on one portion of the rock sample and the concentration of the radiogenic argon is measured on another portion of the same sample. The potassium is usually measured as total potassium and the amount of ^40^K is calculated from the ratio ^40^K/K = 1.167 × 10^−4^, which is known and a constant for all natural materials. The argon is normally determined by fusing the sample in an ultrahigh vacuum system to extract the argon. Isotopic analysis of the extracted argon is performed by mass spectrometry from which the concentration of ^40^Ar^*^ can be calculated after making a correction for atmospheric ^40^Ar. Assuming that all nonradiogenic argon is atmospheric, the correction is calculated using the measured concentration of ^36^Ar in the sample and the ratio ^40^Ar/^36^Ar for atmospheric argon (currently recommended value, 295.5). Finally, the age for the rock sample is calculated using the known decay constant for ^40^K and the measured concentrations of ^40^K and ^40^Ar^*^. Additional details concerning the K-Ar dating technique are given in Ref. [2].

In the ^40^Ar/^39^Ar method, no quantitative measurements of ^40^K and ^40^Ar^*^ are made on the sample. Instead, the age is derived from measurements of isotopic ratios of argon in the sample after irradiation in a nuclear reactor where fast neutrons convert a portion of the ^39^K in the sample to ^39^Ar by an n, p reaction. The converted argon (^39^Ar_K_) is proportional to the ^40^K in the sample since the ^40^K/^39^K ratio is a known constant in nature. The apparent age of the unknown sample can be expressed in terms of the sample’s ^40^Ar^*^/^39^Ar_K_ ratio and an irradiation parameter *J*, which depends on the duration of the irradiation, the neutron flux, and the neutron capture cross section. Because of the difficulty of accurately measuring the fast-neutron dose, a mineral standard or monitor of accurately known K-Ar age is irradiated together with the unknown to monitor the dose. The *J* parameter can be determined by measuring the ^40^Ar^*^/^39^Ar_K_ ratio in the gas extracted from the monitor and is then used along with the ^40^Ar^*^/^39^Ar_K_ ratio measured on the unknown sample to determine its age relative to the monitor. As with the K-Ar dating technique, corrections for atmospheric argon must also be made for ^40^Ar/^39^Ar age measurements. However, the latter technique offers a significant improvement in relative analytical precision since only ratios of argon isotopes need to be measured and therefore it can be used on much smaller samples, which need not be completely outgassed. A comprehensive review of the ^40^Ar/^39^Ar dating technique is given in Ref. [1].

### A2. Summary of Calibration Results for System Volumes

**Table A1:** Measured values for the reference and reservoir volumes and their associated intermediate volumes in the calibration system. Estimates of the combined (*k* = 2) uncertainties in these values are also given.

System	Volume ID	System volume			Associated intermediate volume		
		Volume @23 °C	Combined uncertainty		Volume @23 °C	Combined uncertainty	
		(cm^3^)	(cm^3^)	(%)	(cm^3^)	(cm^3^)	(%)
Calibration	Large Reference	939.642	0.022	0.002	4.6436	0.0017	0.037
	Small Reference	16.5012	0.0021	0.013			

^38^Ar	Reservoir V039	944.69	0.17	0.018	41.858	0.008	0.020
	Reservoir V040	909.25	0.17	0.018			

^40^Ar:^36^Ar 1:1	Reservoir V042	948.08	0.34	0.036	125.019	0.043	0.035
^40^Ar:^36^Ar 30:1	Reservoir V041	3020.13	0.71	0.023			
^40^Ar:^36^Ar 300:1	Reservoir V046	2951.46	0.75	0.026			
Atmospheric Ar	Reservoir V043	3022.51	0.73	0.024			

**Table A2:** Measured values for the Dörflinger pipette volumes and associated combined (*k* = 2) uncertainties. Differences between the nominal values measured by the manufacturer and those measured in the present work are also given.

System	Pipette ID	Nominal volume	Measured volume @23 °C	Combined uncertainty		Nominal - Measured	
		(cm^3^)	(cm^3^)	(cm^3^)	(%)	(cm^3^)	(%)
^38^Ar	U039	0.1011	0.099714	0.000033	0.033	0.00139	1.39
	U040	0.1002	0.099451	0.000033	0.033	0.00075	0.75

^40^Ar:^36^Ar 1:1	U042	0.1011	0.100361	0.000089	0.089	0.00074	0.74
^40^Ar:^36^Ar 30:1	U041	0.2991	0.29701	0.00016	0.053	0.00209	0.70
^40^Ar:^36^Ar 300:1	U046	0.098	0.097086	0.000077	0.080	0.00091	0.94

Atmospheric Ar	U043	0.2986	0.29556	0.00014	0.046	0.00304	1.03
